# Engineering exosomes and biomaterial-assisted exosomes as therapeutic carriers for bone regeneration

**DOI:** 10.1186/s13287-023-03275-x

**Published:** 2023-03-29

**Authors:** Ye Lu, Zizhao Mai, Li Cui, Xinyuan Zhao

**Affiliations:** 1 Stomatological Hospital, School of Stomatology, Southern Medical University, 510280 Guangzhou, China; 2 School of Dentistry, University of California, Los Angeles, Los Angeles, CA 90095 USA

**Keywords:** Exosomes, Mesenchymal stem cells, Preconditioning, Exosome engineering, Biomaterial-assisted exosomes, Bone regeneration

## Abstract

Mesenchymal stem cell-based therapy has become an effective therapeutic approach for bone regeneration. However, there are still limitations in successful clinical translation. Recently, the secretome of mesenchymal stem cells, especially exosome, plays a critical role in promoting bone repair and regeneration. Exosomes are nanosized, lipid bilayer-enclosed structures carrying proteins, lipids, RNAs, metabolites, growth factors, and cytokines and have attracted great attention for their potential application in bone regenerative medicine. In addition, preconditioning of parental cells and exosome engineering can enhance the regenerative potential of exosomes for treating bone defects. Moreover, with recent advancements in various biomaterials to enhance the therapeutic functions of exosomes, biomaterial-assisted exosomes have become a promising strategy for bone regeneration. This review discusses different insights regarding the roles of exosomes in bone regeneration and summarizes the applications of engineering exosomes and biomaterial-assisted exosomes as safe and versatile bone regeneration agent delivery platforms. The current hurdles of transitioning exosomes from bench to bedside are also discussed.

## Introduction

Bone defects are often the consequence of high-energy trauma, infection, tumor excision or congenital defects. In addition, they are frequently accompanied by soft tissue damage, including injuries to muscles, tendons and joints. Bone healing might be initiated immediately following bone loss, which is a highly complex, well-orchestrated regenerative process of forming new bones and is involved in a series of fundamental cellular and molecular events [1]. Unfortunately, the intrinsic regenerative capacity of bone tissues fails to repair massive bone defects above a critical size [2]. Large bone defects require additional clinical intervention, and multiple options are currently available in clinical practice. The defective area is generally reconstructed and filled with grafts of an autogenous, allogeneic, or alloplastic nature to achieve satisfactory bone regeneration [3, 4]. Despite the clinical success of bone grafting, it is limited by major drawbacks, such as invasive surgical procedures, pain, secondary complications and disease transmission [5]. In addition, an ideal bone graft substitute should possess the following characteristics: osteoconduction, osteoinduction, osteoincorporation, osteointegration and osteogenesis [3]. However, to date, no single material has all the necessary properties to efficiently promote bone regeneration. In recent decades, MSC-based tissue engineering has become an effective therapeutic approach in the field of regenerative medicine, which has opened a new path for bone repair and regeneration [6, 7].

MSCs hold great promise for wide-ranging clinical applications and regenerative medicine, as they retain the potential to differentiate into multilineages under defined conditions and the ability to self-renew [8, 9]. In addition, MSCs can be expanded rapidly and easily in vitro, and the supernatants, growth factors and cytokines derived from MSCs are also cell-free sources for tissue regeneration [10]. It has been well documented that MSCs are of great importance during the bone formation or healing process [11, 12]. They have the capacity of migration and homing into or around the injured sites, which are the essential steps for achieving bone repair and regeneration. Then, MSCs are able to commit to osteogenic/chondrogenic progenitors and eventually differentiate into osteocytes/chondrocytes for bone tissue reconstruction after bone defects [13]. In addition, due to the immunomodulatory properties of MSCs, they escape immune recognition and protect against the cytotoxic effects of the host’s immune system [14]. Moreover, MSCs might promote bone regeneration by modulating the microenvironment. For instance, human adipose tissue-derived MSCs (hASCs) and their conditioned medium (CM) enhanced bone regeneration in a rabbit model of surgical bone lesions, suggesting that hASCs promoted bone regeneration mainly by releasing paracrine factors [15].

Considering the critical role of MSCs in bone regeneration, as expected, MSCs have been shown to be an efficacious treatment for bone defects [16]. For example, transplanted MSCs have been shown to not only survive and proliferate in injured sites but also differentiate into osteoblasts, leading to bone tissue reconstruction in a model of osteonecrosis of the femoral head [17]. Similarly, bone marrow MSC (BMSC) transplantation significantly accelerated bone regeneration in a rat mandibular osteodistraction model [18]. Percutaneous injection of MSCs isolated from bone marrow alone or with bioscaffolds such as biphasic hydroxyapatite/β-calcium-triphosphate granules has been shown to promote bone repair in patients with delayed healing of long bone fractures or avascular necrosis of the femoral head [19, 20]. Despite the vast number of MSC-based clinical trials that have been completed, none of the approaches have been approved for the treatment of bone defects. There are several significant barriers to the clinical application of MSC-based bone regeneration [21]. First, standardized protocols need to be established to avoid unwanted MSC differentiation during ex vivo expansion [22]. Second, the beneficial effects of MSC osteogenic differentiation for bone repair and regeneration are significantly impaired due to the poor engraftment and survival of MSCs in the injury sites [23]. Third, the number of administered MSCs is not sufficient for treating large bone defects in the clinical setting, and long-term in vitro expansion might affect the biological functions of MSCs [24]. Other limitations in MSC-based bone regeneration concern ethical issues and the possible risk of tumorigenicity [25].

The key success factor of MSC-based bone regeneration is because MSCs are able to differentiate into bone-forming osteoblasts and cartilage-forming chondrocytes [26]. It is expected that the implanted or injected MSCs will commit to a particular lineage at the site of injury for tissue repair and regeneration. However, the survival status and the differentiation capacity of the implanted/injected MSCs are unfavorable, thereby casting doubt on its correct mechanism of action. Interestingly, growing evidence has demonstrated that MSCs might exert their therapeutic effects mainly through paracrine effects [19, 27, 28]. The MSC-derived secretome includes vesicles, growth factors, cytokines, chemokines, extracellular matrix (ECM) and metabolites and plays an indispensable role in modulating tissue repair and regeneration [29]. For example, after incorporating human umbilical cord MSC (hucMSC)-derived extracellular vesicles into tubular epithelial cells, the complex was injected into the injured kidneys, and surprisingly, kidney function and morphology were significantly improved [30]. MSC-derived immunomodulators and trophic factors, including vascular endothelial growth factor (VEGF), transforming growth factor-β, and hepatic growth factor, contribute to the positive effects of the BMSC secretome in the bone regenerative process [31]. The secretome in the CM from MSCs mimicked the native bone healing procedures to enhance bone regeneration [6].

As this review aims to summarize the recent progress on engineering exosomes and biomaterial-assisted exosomes as therapeutic carriers for bone regeneration, we mainly referred to the research articles published in recent 5 years. The databases we mainly used included PubMed, Google scholar, Scopus, Web of Science and ProQuest by using the following keywords: “exosomes”, “mesenchymal stem cells”, “preconditioning”, “exosome engineering”, “biomaterial scaffolds”, and “bone regeneration”. The articles were further screened to identify their relevance to this review.

## Exosome concept, biogenesis, content and application

**Fig. 1:**
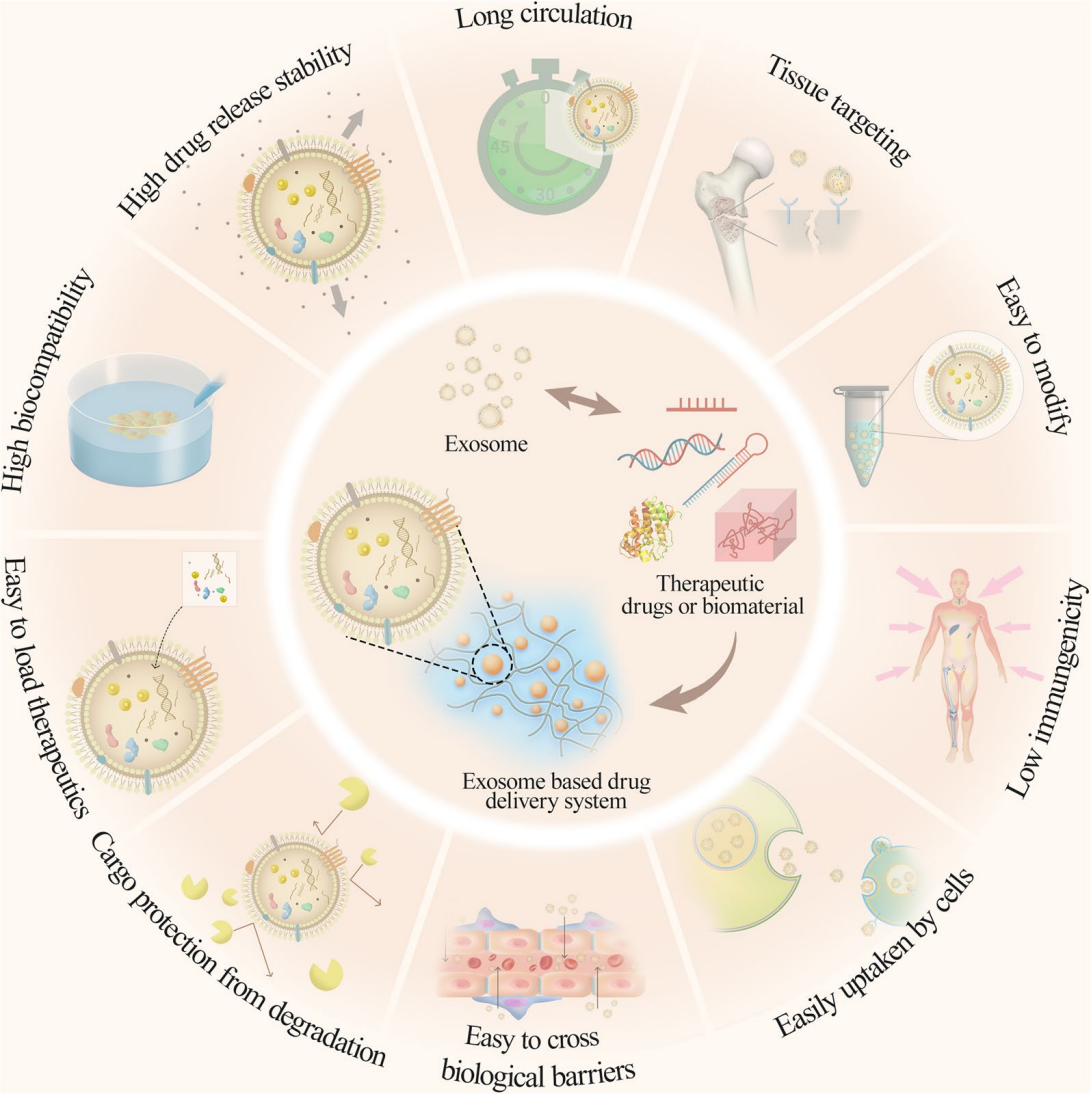
Exosomes derived from MSCs as a delivery vehicle have the advantages of low immunogenicity, easily uptaken by cells, easily crossing biological barriers, cargo protection from degradation, easily loading therapeutics, high cargo release stability, high biocompatibility, long circulation, and having the ability of tissue targeting. Therefore, they have been developed as a novel strategy for treating various diseases

Exosomes are nanosized, lipid bilayer-enclosed structures carrying proteins, lipids, RNAs, metabolites, growth factors and cytokines and have attracted great attention for their critical role in mediating cell–cell communication locally and systemically [32]. Exosomes are derived from the endocytic pathway of most cells and released from multivesicular bodies (MVBs) into nearly all biological fluids, such as blood, saliva and urine [33]. The formation of exosomes can be classified into four processes: invagination, endosome formation, fusion and secretion. Initially, early endosomes are formed by invagination of the plasma membrane, which are subsequently transformed into late endosomes containing MVBs. Following the fusion of MVBs with the plasma membrane, the exosomes are consecutively released from the cells [34]. The abundance, composition and functional properties of exosomes depend on the origin of the cell/tissue, the physiological/pathological state and even the microenvironment in which the parental cells reside [35]. The main role of exosomes lies in their capability to deliver information to adjacent cells and even to those cells that are remote from the exosome cellular origin, thereby influencing their function [36]. The contents of circulating exosomes are different between patients and healthy individuals, which can reflect the real-time state. Therefore, exosomes are emerging as robust and promising noninvasive biomarkers for the diagnosis, prognosis and prediction of treatment efficacy in various diseases [37]. More importantly, exosomes have been developed as therapeutic carriers for treating diseases. The advantage of using exosomes as delivery vehicles is their capacity to load both hydrophobic and hydrophilic items either inside or in the lipid bilayer [38]. The cargos are shielded by the bilayer membranes, which effectively protects the cargos from enzymatic degradation [38, 39]. In addition, the long circulation property of MSC exosomes prolongs their residence time in the blood and reduces the clearance rate of drugs they carry. MSC-produced exosomes can target specific cell types through their surface receptors [40, 41]. Exosomes have also been proven to be able to cross biological barriers and travel into deep tissues [40, 42]. Moreover, exosomes are efficacious for promoting regeneration, inducing stem cell differentiation and triggering particular immunological reactions [43]. Furthermore, they also exhibit great biocompatibility, biodegradability and stability, as well as low immunogenicity (Fig. 1) [43]. Small molecules, nucleic acid drugs or bioactive molecules can be functionally incorporated into exosome-based nanocarriers and then transported to targeted sites to achieve therapeutic outcomes [44]. Pascucci et al. [45] reported that MSC-derived exosomes loaded with chemotherapeutic drugs trafficked to tumor tissues and exerted antitumorigenic effects. In addition, MSC-derived exosomes also exhibit promising therapeutic potential for bone regeneration. As a critical mediator of intercellular communication, exosomes play a prominent role in a variety of biological processes including bone formation. MSC-derived exosomes exert prolific therapeutic efficacy in bone defects as they are capable of targeting bone tissues to induce osteogenic differentiation, thereby accelerating the process of bone regeneration [46]. For example, in a rat model of disuse osteoporosis, the diseased animals treated with hucMSC-derived exosomes formed new bone, and the bone structural parameters were significantly improved [47]. Exosomes secreted from human-induced pluripotent stem cells (hiPSC) enhanced bone regeneration in the osteoporotic rat model [48]. BMSC-derived exosomes stimulate osteoblast proliferation and osteogenic differentiation, thereby promoting osteogenesis in the rabbit model of osteonecrosis of the femoral head [49]. In addition, exosomes have a certain degree of natural targeting capacity. The targeting specificity of exosomes is mainly achieved by surface receptor–ligand binding to target specific cells, and then, exosomes can stimulate intracellular signaling in recipient cells or deliver their components to stimulate osteoblast proliferation and differentiation [50]. However, the therapeutic efficacy of natural exosomes for bone regeneration is limited [51]. MSC-derived exosomes can be captured by the reticuloendothelial system or cleared by the mononuclear phagocyte system, which might prevent their accumulation in the injured sites to exert desirable regenerative effects [52, 53]. Additionally, the natural targeting property of MSC exosomes is not accurate enough for delivering the indicated cargos to the specific recipient cells or tissues. Engineering or manipulating exosomes can optimize their targeting capability, providing potential wide clinical applications for treating diseases, including bone defects [54]. Here, we summarize recent research progress on naturally derived exosomes and engineered exosomes in bone repair and regeneration. In addition, as biomaterial scaffolds are the basic material for bone tissue engineering, the effects of biomaterial-assisted exosomes on bone regeneration have also been reviewed. The practical challenges associated with exosome-based bone regeneration are also discussed.

## Mechanism of MSC-derived exosomes for bone regeneration

MSCs-derived exosomes promote bone regeneration by directly transferring their internal cargos and subsequently controlling downstream signaling pathways in the targeted cells [55, 56]. Exosomes also regulate immune responses, inhibit osteoclast activities and induce osteogenesis and angiogenesis [57]. Various signaling cascades, including BMP/Smad, Wnt/β-catenin and PI3K/AKT, are activated by MSC exosomes, resulting in bone regeneration by promoting osteoblast proliferation and differentiation as well as the recruitment of endogenous MSCs to bone defect sites [57–59]. In addition, MSC exosomes repair bone defects by enhancing local angiogenesis and suppressing bone resorption, and activation of AKT/mTOR signaling pathway might be responsible for the osteogenesis-promoting effects [60, 61]. Moreover, MSC exosomes are effective in improving overall wound healing by inhibiting inflammatory responses and preventing osteoclast activities [62, 63]. The release of BMP-2 and other osteogenic growth factors from macrophages and non-stem cells were markedly elevated following the addition of MSC exosomes, which subsequently accelerated the healing of damaged tissues [46, 62]. It has also been demonstrated that MSC exosomes promote angiogenesis in bone defect area, which play a crucial role in bone regeneration [60, 61]. Vasculature serves as the main transport conduit for hormones and growth factors to supply the bone defect area with oxygen, nutrients and metabolites, which are essential for bone growth and regeneration [64]. A number of exosomal miRNAs such as miRNA-135b, miRNA-204 and miRNA-196a have been shown to play a critical role in regulating the bone regeneration process [65–67]. Interestingly, the immune microenvironment formed by immune cells and their metabolites is also indispensable for bone regeneration. The immune microenvironment not only controls the activities of osteoblasts and osteoclasts, but also the secretion of chemokines, growth factors and inflammatory factors, which in turn modulates the formation of new bone and the strength of existing bones [68, 69]. MSC exosomes are crucial in modulating immune cell activities in the complicated internal environment [46, 62]. For instance, by delivering the intracellular cargos, MSC exosomes induce the polarization of macrophages from the M1 to the M2 phenotype, contributing to the establishment of anti-inflammatory microenvironment during bone defect repair [70].

## Modification of MSC-derived exosomes for bone regeneration

**Fig. 2:**
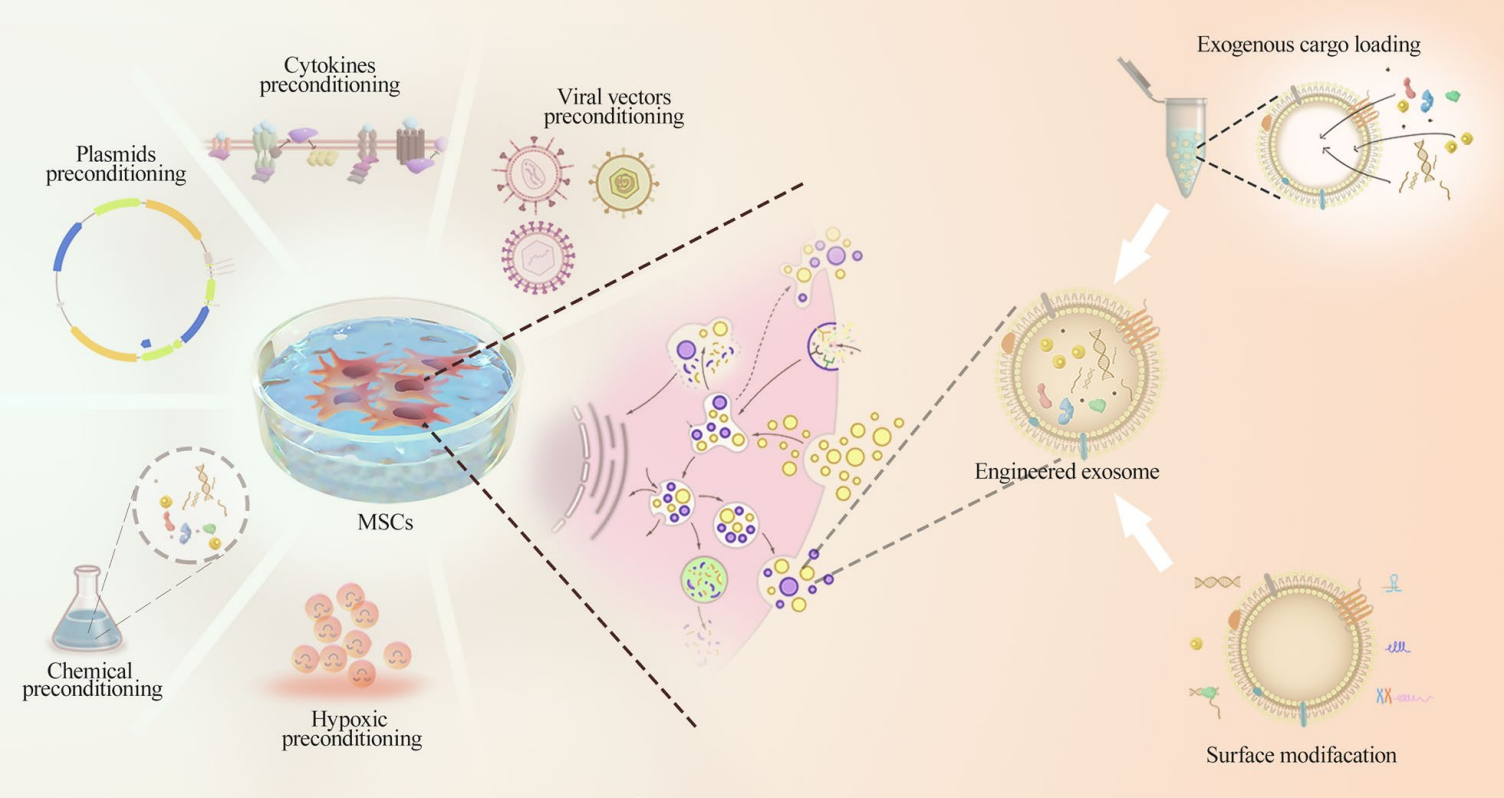
The avenues for the modification of exosomes are mainly divided into two aspects: preconditioning of parental cells and exosome engineering. Preconditioning of parental cells for bone regeneration mainly included hypoxic preconditioning, cytokine preconditioning and chemical preconditioning. Exosome engineering is mainly divided into two categories: cargo packaging into exosomes and surface modification of exosomes

Due to the limitations of natural exosomes, such as lack of sufficient production, instability in circulation and poor targeting capacity, various strategies have been developed to modify MSC-derived exosomes and enhance their therapeutic potential [71]. The avenues for the modification of exosomes are mainly divided into two aspects: preconditioning of parental cells and exosome engineering (Fig. 2).

## Preconditioning of parental cells

Although exosomes derived from MSCs have been widely explored for cell-free therapy of various diseases, limitations, including low production of MSC exosomes, the demand for large amounts of exosomes, heterogeneity and low targetability impede the clinical trials of MSC-derived exosomes [51]. Growing evidence indicates that the preconditioning of parental cells is an adaptive strategy to enhance the therapeutic efficacy and yield of MSC exosomes [51]. Preconditioning of MSCs overcomes the limitations of natural exosomes by enhancing MSC paracrine activities to increase the production of MSC exosomes [72, 73]. In addition, pretreatment can promote exosomes from MSCs to regulate both the innate and adaptive immune responses of recipient cells [72, 73]. The approaches for preconditioning of MSCs mainly include hypoxic preconditioning, cytokine preconditioning and chemical preconditioning.

### Hypoxic preconditioning

Hypoxia refers to the cell culture conditions under 0–10% oxygen tension. Oxygen tension plays an essential role in maintaining bone homeostasis [74]. The 1% oxygen tension in physiological cartilage and bone marrow is far lower than the 21% O_2_ routinely used for MSC culture [75]. A hypoxic environment can lead to cell death; however, hypoxic preconditioning can reduce hypoxia-induced apoptosis by elevating the expression of pro-survival signaling [76]. Interestingly, recent studies showed that the therapeutic effects of MSC-derived exosomes after hypoxic preconditioning were significantly enhanced for treating a wide array of diseases, including bone fracture healing [77, 78], acute kidney injury [79], spinal cord injury [80], diabetic wounds [81], myocardial infarction [82–84] and insufficient vessel growth [85].

Hypoxic preconditioning can enhance the cytoprotective and regenerative effects of MSCs to promote the secretion and therapeutic effects of their exosomes in injury sites such as bone defects and osteoarthritis [86, 87]. Mechanistically, hypoxic preconditioning increases the levels of cytoprotective molecules and maintains the multipotent and proliferative capabilities of stem cells [88–90]. An increasing number of studies have demonstrated that hypoxic preconditioning may improve the osteogenic differentiation and proliferation of MSCs by elevating the expression of growth factors and stemness-related markers such as SOX2, OCT4, NANOG and KLF4 [91, 92]. In addition, hypoxia preconditioning contributes to the activation of hypoxia-inducible factor (HIF-1α), which subsequently promotes the expression of angiogenic factors [93]. Similarly, hypoxic preconditioning can increase the relative expression levels of molecule contained in MSC exosomes in the same way.

MSC-derived exosomes from hypoxic conditioning have shown superior abilities in enhancing osteogenesis and angiogenesis, and the secretion of exosomes was found to be significantly improved after hypoxic preconditioning [77, 78]. For example, the focal adhesion pathway, thyroid hormone synthesis and VEGF signaling pathway were significantly upregulated in exosomes from stem cells from human-exfoliated deciduous teeth (SHEDs) after hypoxic preconditioning [77]. Thus, exosomes derived from hypoxic-preconditioned SHEDs showed superior potential for cellular osteogenesis and angiogenesis in a rat calvarial defect model [77]. In addition, exosomes derived from hypoxia-preconditioned hucMSC healed bone fractures via the SPRED1/Ras/Erk signaling pathway [78]. In addition, hypoxic preconditioning activated HIF-1α to promote the secretion of exosomal miRNA-126 for bone fracture healing by enhancing proliferation, angiogenesis and migration to a greater extent [78]. Exosomes from BMSCs after hypoxic preconditioning promoted the proliferation and migration of chondrocytes and inhibited the apoptosis of chondrocytes via the miRNA-181c-5p/MAPK or miRNA-18-3p/JAK/STAT signaling pathways [86]. MicroRNAome analysis revealed that hsa-miRNA-18a-3p, hsa-miRNA-181c-5p, hsa-miRNA-337-5p and hsa-miRNA-376a-5p were differentially expressed between normoxia-preconditioned and hypoxia-preconditioned BMSC-derived exosomes [86]. In contrast, oxidative stress caused by H_2_O_2_ inhibited the release of exosomes from human periodontal ligament cells (hPDLCs) through a large accumulation of MVB caused by reducing the expression of Rab11a and Rab27a [94]. In general, hypoxia preconditioning is a promising strategy for enhancing the effectiveness of MSC-exosome-based therapy in bone regeneration.

### Cytokine preconditioning

Accumulating evidence has demonstrated that preconditioning MSCs with inflammatory cytokines promotes the secretion of immunoregulatory factors [95]. The immune responses regulated by exosomes from cytokine-preconditioned MSCs include transferring MSCs to a more anti-inflammatory phenotype and inducing the polarization of anti-inflammatory M2 macrophages [96–99]. MSC-chemokine preconditioning can concentrate numerous immunocytes for their migration and homing into or around the injured sites to combat inflammatory responses. This shows that MSC-chemokine preconditioning is a promising strategy for bone regeneration by decreasing inflammation in bone defects [98–101].

Tumor necrosis factor-alpha (TNF-α) is one of the most studied inflammatory cytokines. An increasing number of studies have shown that TNF-α preconditioning enhances the therapeutic effects of exosomes derived from MSCs in various diseases, including bone defects [100], periodontitis [98], retinal ganglion injury [102], acute liver failure [96] and urethral stricture [97]. TNF-α promoted the secretion of a greater number of anti-inflammatory MSC exosomes by upregulating inflammatory suppression-related miRNAs, including miRNA-146a and miRNA-299-3p [96, 97]. These anti-inflammatory MSC exosomes contributed to the transfer of MSCs to a more anti-inflammatory phenotype [96, 97]. Preconditioning MSCs with TNF-α not only promoted the anti-inflammatory ability of MSC exosomes but also enhanced the osteogenic differentiation potential of MSC exosomes. hASCs preconditioned with TNF-α regulated the proliferation and osteogenic differentiation of human primary osteoblastic cells through Wnt signaling [100]. Exosomes derived from gingival tissue-derived MSCs (GMSCs) preconditioned with TNF-α regulated inflammation and osteoclastogenesis in a ligature-induced periodontitis mouse model by promoting the secretion of exosomes, upregulation of the exosomal CD73 expression, and induction of the polarization of anti-inflammatory M2 macrophages [98]. In addition, preconditioning GMSCs with TNF-α promoted the therapeutic effects of GMSC exosomes, including decreasing the number of tartrate-resistant acid phosphatase (TRAP)-positive osteoclasts and suppressing periodontal bone resorption [98].

Interleukin-1β (IL-1β) has been shown to promote the anti-inflammatory effects of MSC exosomes in osteoarthritis by increasing the expression of miRNA-147b [101]. In addition, exosomes derived from BMSCs preconditioned with IL-1β inhibited the inflammation of hippocampal astrocytes and status epilepticus mice through suppression of the NRF2 signaling pathway [103]. Exosomes derived from transforming growth factor-β1 (TGF-β1)-preconditioned BMSCs attenuated cartilage damage in osteoarthritis rat models by upregulating miRNA-135b, which promoted M2 polarization of synovial macrophages by targeting MAPK6 [99]. In summary, preconditioning of certain cytokines and growth factors promotes the osteogenic potential and anti-inflammatory function of MSC exosomes. It should be pointed out some potential cytokines and growth factors are promising candidates for MSC preconditioning. For example, preconditioning of MSCs with IFN-γ showed the potential of anti-inflammatory and angiogenic abilities [104]. In addition, growth factors such as FGF 2, IGF-1, IGF-2, TGF-β1 and PDGF are efficacious in promoting soft tissue healing, and the effects of MSC preconditioning with these growth factors on bone regeneration warrant further investigation [104, 105].

### Chemical preconditioning

Chemical signals can be rapidly detected by MSCs and may significantly alter their phenotypes [51]. In addition, the influence also changes the secretion and content of MSC exosomes, which show promising potential in immune regulation and tissue regeneration [106]. For example, exosomes derived from kartogenin-preconditioned hucMSCs induced chondrogenic differentiation by promoting the secretion of exosomal miRNA-381-3p through targeting TAOK1 [107]. Similarly, kartogenin-reconditioned mouse BMSC (mBMSC)-exosomes have a better ability to promote chondral matrix regeneration than exosomes derived from untreated mBMSCs [108]. In addition, exosomes derived from curcumin-preconditioned MSCs attenuated osteoarthritis by modulating the miRNA-124/NF-kB and miRNA-143/ROCK1/TLR9 signaling pathways [109].

## Exosome engineering

Exosome engineering has been explored to enhance the therapeutic potential of MSC exosomes [110]. Exosome engineering is mainly divided into two categories: cargo packaging into exosomes and surface modification of exosomes [51].

### Cargo packaging into exosomes

Packaging cargo into exosomes can promote the therapeutic effects of MSC exosomes. The methods of packaging cargo into exosomes can be divided into two main categories: endogenous and exogenous cargo loading methods [51]. Endogenous cargo loading is the modification of parental cells with viral vectors and plasmids, while exogenous cargo loading is the direct loading of drugs into the extracted MSC exosomes [51]. Endogenous cargo methods are usually used to load endogenous proteins and nucleotides with therapeutic effects, while exogenous cargo loading methods are usually used to load small molecule drugs [111].

### Endogenous cargo loading

Accumulating evidence shows that the functional biomolecules of MSC exosomes, such as nucleotides and proteins, play essential roles in osteogenesis, angiogenesis, immunomodulation and tissue regeneration in different kinds of disease models [112]. Genetic engineering tools, such as viral vectors and plasmids, can genetically manipulate the endogenous molecule expression levels in MSCs.

Growing evidence indicates that miRNA delivery plays a crucial role in improving the therapeutic potential of MSC exosomes in various disease models. Exosomes from miRNA-375-overexpressing hASCs embedded with hydrogel promoted the bone regeneration of BMSCs by inhibiting the expression of IGFBP3 [113]. Exosomes from miRNA-181b-overexpressing BMSCs enhanced osteogenesis in vitro and osteointegration in vivo by secreting VEGF and bone morphogenetic protein 2 (BMP2) to promote M2 polarization and inhibit inflammation by activating the PRKCD/AKT signaling pathway [114]. Exosomes from miRNA-140-5p-overexpressing synovial mesenchymal stem cells promoted the migration and proliferation of articular chondrocytes in an osteoarthritis rat model [115]. Exosomes from BMP2-overexpressing BMSCs enhanced osteogenic regeneration and osteoinduction in a rat calvarial defect model [116, 117]. Exosomes derived from BMSCs modified with mutant HIF-1α (BMSC-Exos^MU^) significantly promoted the osteogenic differentiation ability of BMSCs in vivo, while exosomes derived from BMSCs modified with wild-type HIF-1α (BMSC-Exos^WT^) did not show osteogenic differentiation ability [118]. In addition, BMSC-Exos^MU^ markedly increased angiogenesis and bone regeneration in the necrotic regions by increasing microvascular density and trabecular reconstruction [118].

### Exogenous cargo loading

Physical methods are usually employed to encapsulate drugs into engineered exosomes, such as permeabilization with saponins, freeze‒thaw cycles, extrusion, or sonication, for treating various diseases [119, 120]. For example, sonication was used to fractionate exosome membranes into small vesicles, and then, drugs were extruded into the vesicles [119, 120]. Other methods for exogenous cargo loading usually included permeabilization with saponin, freeze‒thaw cycles and incubation at room temperature for treating various diseases [119]. BMP2 was directly loaded into engineered extracellular vesicles of BMSCs, which induced osteogenic regeneration in vitro and in vivo, while BMP2 was absent in the exosomes of untreated group [121]. Compared to naturally occurring extracellular vesicles, engineered BMP2-extracellular vesicles protected BMP2 from proteolysis [121]. ATDC5 was encapsulated with the VEGF gene into exosomes, which induced vascularized bone regeneration [122]. Mechanistically, exosomes loading VEGF gene promoted the osteogenic differentiation ability of MSCs by controllably releasing the vascularized gene VEGF to remodel the vascular system and combining the 3D-printed porous bone scaffolds with engineered exosome nanoparticles (NPs) [122]. In addition, the exosomes encapsulated with the VEGF gene showed the best bone regeneration ability and induced the formation of a large amount of new bone in a rat radial defect repair model [122].

## Surface modification

Surface modification is used to enhance the targeting ability of MSC exosomes to promote their therapeutic effects by direct modification of exosome surface molecules [111]. Targeting ligands can be added onto the surface of exosomes to precisely deliver therapeutic drugs to the indicated lesions. For example, an aptamer was combined with exosomes derived from bone marrow stromal cells (STExos) to generate the STExo-aptamer complex [123]. The complex enhanced the bone mass in an ovariectomy-induced postmenopausal osteoporosis mouse model and promoted bone healing in a femur fracture mouse model after intravenous injection [123]. C-X-C motif chemokine receptor 4 (CXCR4) was added to the surface of exosomes derived from genetically engineered NIH-3T3 cells [124]. The complex was then combined with liposomes carrying antagomir-188 to generate hybrid NPs. The hybrid NPs recovered the loss of age-related trabecular bone and reduced cortical bone porosity in mice by specifically gathering in the bone marrow and controllably releasing antagomir-188 to inhibit adipogenesis and promote osteogenesis of BMSCs [124].

Collectively, the modification of exosomes enhances the osteogenic and angiogenic potential of MSC exosomes by increasing the secretion of MSC exosomes and regulating the expression levels of osteogenic and angiogenic genes, which are promising therapeutic strategies for bone regeneration. We summarized the methods of exosome modification in Table 1.

**Table 1:** Modification of exosomes for improving osteogenic therapy potential

Modification	Source	Model and time	Dose and route	Molecular mechanism	References
Hypoxic preconditioning	SHEDs	Rat calvarial defects model, 4 and 8 weeks	NA, defect sites injection	Focal adhesion, thyroid hormone synthesis and VEGF signaling pathway ↑	[50]
	hucMSCs	Mice bone fracture model, 7 days	200 μg, fracture sites injection	miR-126 expression ↑; SPRED1/Ras/Erk signaling pathway ↑	[51]
	BMSCs	Rat osteoarthritis model, 8 weeks	100 μg, articular cavity injection	hsa-miR-181c-5p, hsa-miR-18a-3p, hsa-miR-376a-5p, and hsa-miR-337-5p expression ↓	[65]
	hPDLCs	NA	NA	Rab11a and Rab27a expression ↓	[66]
TNF-α	hASCs	NA	NA	Wnt-3a content ↑	[72]
IL-1β	BMSCs	NA	NA	NRF2 signaling pathway ↓	[73]
TGF-β1	BMSCs	Rat osteoarthritis model, NA	1 × 10^10^, articular cavity injection	miR-135b expression ↑	[71]
Kartogenin	hucMSCs	Rabbit cartilage defects model, 4 weeks	1.0 × 10^7^ cells/mL, defect sites injection	miR-381-3p expression ↑	[77]
	mBMSCs	Rat osteoarthritis model, 4, 6 and 8 weeks	NA, articular cavity injection	Chondral matrix regeneration ↑	[78]
Curcumin	BMSCs	Mice osteoarthritis model, NA	NA, articular cavity injection	miR-143 and miR-124 expression ↑	[79]
Endogenous cargo loading	miR-375-overexpressing hASCs	Rat calvarial defects model, 3 days, 2, 4 and 8 weeks	50 μg/mL, defect sites injection	IGFBP3 ↓	[83]
	miR-181b-overexpressing BMSCs	Rat femoral defects model, 3 months	NA, defect sites injection	Macrophage M2 polarization ↑; PRKCD ↓	[84]
	miR-140-5p-overexpressing synovial mesenchymal stem cells	Rat osteoarthritis model, 12 weeks	10^10^, articular cavity injection	YAP ↑	[85]
	BMP2-overexpressing BMSCs	Rat calvarial bone defects model, 4, 8 and 12 weeks	5 × 10^8^, defect sites injection	Osteoinductive growth factors (BMP2, BMP9) and transcription factors (RUNX2, OSX) ↑	[86]
	BMP2-overexpressing BMSCs	Mice femoral defects model, 15 and 30 days	50 μg, defect sites injection	BMP2/Smad signaling pathway ↑	[87]
	BMSC-Exos^MU^	Rabbit steroid-induced avascular necrosis of femoral head model, 6 weeks	80 μg/mL, necrosis region injection	Osteocalcin ↑; alkaline phosphatase ↑	[88]
Loading BMP2	BMSCs	Mice muscle pocket model, 4 weeks	150 ng, inserting into the pocket	BMP2, RUNX2 expression ↑	[91]
Loading VEGF	ATDC5	Rat segmental defects model, 6 weeks and 12 weeks	10 μg, implantation	Alkaline phosphatase ↑; COL1A1 ↑	[92]
Aptamer	bone marrow stromal cells	Mice postmenopausal osteoporosis model, 2 months	100 μg, intravenous injection	miR-26a expression ↑	[93]
CXCR4	NIH-3T3 cells	Mice age-related osteoporosis model, 8 weeks	100 μL, tail vein injection	miR-188 expression ↓	[94]

## Biomaterial-based MSC-derived exosomes for bone regeneration

**Fig. 3:**
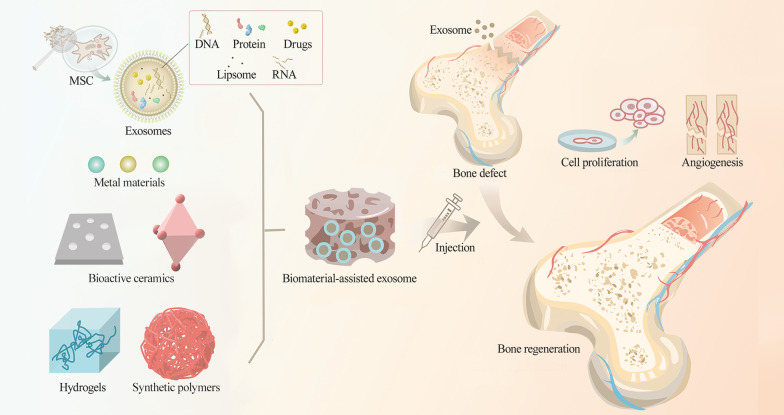
Exosomes can bind to biomaterial scaffolds, including metal materials, bioactive ceramics, hydrogels and synthetic polymers, and then target injured sites to enhance bone regeneration mainly by inducing osteogenesis and angiogenesis

Despite advancements in natural exosomes for bone regeneration applications, there are still certain limitations in complex composition properties, limited cargo loading efficiency and undesirable retention stability. However, exosomes can bind to biomaterial scaffolds to enhance bone regeneration mainly by inducing osteogenesis and angiogenesis (Fig. 3). A range of biomaterial scaffolds endows exosomes with desirable pharmaceutical acceptability by extending exosome storage time and altering the release properties, thereby overcoming the drawbacks of natural exosomes. The use of biomaterial-assisted exosomes has proven to be a novel strategy for regenerating bone tissues [125]. Biomaterials employed for scaffolds can be divided into metal materials, bioactive ceramics, hydrogels and synthetic polymers. We have summarized the biomaterial-based exosomes for bone regeneration applications in Table 2.

**Table 2:** Biomaterials scaffold for bone regeneration application

Biomaterials scaffold	Source	Model and time	Dose and route	Results	References
Silver NPs	MSCs	NA	NA	Osteogenic differentiation of hBMSCs ↑	[97]
3D printed titanium alloy	hMSCs	Rat radial bone defects model, 4 and 12 weeks	NA, implantation with scaffolds	Osteogenic miRNAs ↑; osteogenic differentiation pathways (PI3K/Akt and MAPK) ↑; anti-osteogenic miRNAs ↓	[98]
MBG	BMSCs	Rat critical-sized calvarial defects model, 12 weeks	2 × 10^11^/mL, implantation with scaffolds	Osteogenic-related marker expression ↑	[101]
β-TCP	MSCs	Rat critical-sized calvarial defects model, 8 weeks	5 × 10^11^/mL (100 μL) and 1 × 10^12^/mL (100 μL), implantation with scaffolds	Osteogenic differentiation pathway (PI3K/AKT) ↑	[103]
β-TCP	hiPSCs	Rat osteoporotic defects model, 8 weeks	100 µg and 200 μg, implantation with scaffolds	Osteogenesis and angiogenesis ↑	[104]
β-TCP	BMSCs	Rat cranial defects model, 12 weeks	200 μg, implantation with scaffolds	Transcription factor in bone formation (RUNX2) ↑	[105]
β-TCP	SHEDs	Rat periodontal bone defects model, 4 weeks	2 μg/μL, implantation with scaffolds	Osteoblastic induction ↑	[106]
β-TCP	hPDLSCs	Rat periodontal bone defects model, 8 weeks	150 µg/µL, implantation with scaffolds	Osteogenic differentiation pathway (Wnt) ↑	[107]
NC bone filler	MSCs	Rat tibia defects model, 8 weeks	10 μg, implantation with scaffolds	Tissue mineralization ↑	[108]
HA-Gel/nHP	uMSCs	Rat critical-sized cranial defects model, 4 and 8 weeks	200 μL, implantation with scaffolds	Osteogenic factors ↑	[112]
HA-ALG/HAP	hucMSCs	Rat critical-sized calvarial bone defects model, 8 weeks	1 μg/μL (250 μL), implantation with scaffolds	Osteogenic and osteoinductive abilities ↑	[113]
CS/β-glycerophosphate	BMSCs	Rat calvarial bone defects model, 12 weeks	1 × 10^8^/mL, implantation with scaffolds	Osteogenesis and angiogenesis ↑	[114]
Gelatin	hASCs	Rat skull defects model, 4 and 8 weeks	0.8 mg, implantation with scaffolds	Transformation of M1 macrophages to M2 macrophages ↑; inflammatory response ↓; bone healing↑	[115]
CS	DPSCs	Mice periodontitis model, NA	50 μg, periodontal injection	miR-1246 expression ↑; modulating macrophages from pro-inflammatory phenotype to an anti-inflammatory phenotype	[117]
SIS/CA/peptides	BMSCs	Rat skull defects model, 12 weeks	1 μg/μL (300 μL), implantation with scaffolds	Osteogenesis-related genes (RUNX2) ↑	[118]
PEG/DNA	SCAPs	Rat mandibular alveolar bone defects model, 2 and 6 weeks	NA, local injection	Osteogenesis-related miRNAs ↑	[119]
CHA/SF/GCS/DF-PEG	hucMSCs	Rat femoral condyle defects model, NA	50 μg, implantation with scaffolds	BMP-2 expression ↑	[120]
Mg/GA/MOF	hBMSCs	Rat calvarial defect model, 10 weeks	40 μg/mL, implantation with scaffolds	Osseointegration ↑; anti-inflammatory abilities ↑	[121]
PEGMC/β-TCP	rBMSCs	Rat posterolateral spinal fusion model, NA	100 μg/mL, implantation with scaffolds	Osteogenesis and vascularization ↑	[122]
PLGA/pDA	hASCs	Mice skull defects model, 6 weeks	25 µg/mL, implantation with scaffolds	Osteoblastogenesis-related genes (RUNX2, ALP and COL1A1) ↑; osteocalcin ↑	[123]
PLGA/PEG	hDPSCs	Mice calvarial bone defects model, 8 weeks	NA, implantation with scaffolds	Bone tissue neogenesis ↑	[124]
PLA	MSCs	NA	NA	Osteogenic differentiation in hBMSCs ↑; inflammation ↓	[125]
Sulfonated PEEK	BMSCs	Rat femoral defects model, 8 weeks	127.39 μg/cm^2^, implantation with scaffolds	Macrophage M2 polarization ↑; osseointegration ↑	[126]

## Metal materials

Biodegradable metal materials are widely used biomaterials in exosome engineering for repairing bone defects. Their excellent biocompatibility, easy production and processing, mild stretchability, and corrosion resistance make them suitable for use as exosome-loaded scaffolds to promote bone regeneration [126]. For example, MSC-derived exosomes incorporated into silver NPs hybrid scaffolds induced osteogenesis, thus serving as a promising cell-free therapeutic for bone regeneration [127]. In addition, titanium alloys are also scaffolds for bone defect treatment with desirable biocompatibility, excellent friction coefficient, high porosity and corrosion resistance. Exosomes derived from human MSCs (hMSCs) loaded into 3D-printed titanium alloy scaffolds can be used to achieve cell-free bone regeneration. The exosomes released by 3D-printed titanium alloy-decorated hMSCs induced osteogenic differentiation of hMSCs and regenerated bone tissues by upregulating osteogenic miRNAs, downregulating anti-osteogenic miRNAs and activating the PI3K/Akt and MAPK signaling pathways [128].

## Bioactive ceramic

Bioactive ceramic biomaterials, including bioactive glass and tricalcium phosphate, are also desirable alternatives for repairing bone tissue that have good toughness and biocompatibility and different characteristics [129].

### Mesoporous bioactive glass

Mesoporous bioactive glass (MBG) is a hierarchical structure scaffold that has been proven to be beneficial for bone repair with positive biological effects on osteogenesis [130]. For example, optimized osteogenic BMSC-derived exosomes were loaded into MBG scaffolds to realize bioactivity maintenance and sustained release of exosomes, thereby efficiently promoting bone-forming ability and enhancing rapid initiation of bone regeneration [131].

### Tricalcium phosphate

β-Tricalcium phosphate (β-TCP), resembling the human autologous bone component, is one of the most commonly used bone tissue engineering biomaterials. β-TCP scaffolds with advanced osteotransductive ability and high osteoinductive potential are able to maintain the balance between material degradation and osteogenesis by releasing calcium ions and sulfates [132]. Therefore, β-TCP is an optimal scaffold for bone regeneration. Porous β-TCP scaffolds can be used as exosome carriers and implanted into the bone defect area, thereby promoting bone regeneration after being loaded into MSC-derived exosomes in a concentration-dependent manner. For instance, exosomes derived from MSCs in combination with β-TCP induced new bone formation. This MSC-exosome-β-TCP scaffold complex improved the osteoinductivity of β-TCP and possessed better osteogenesis activity than β-TCP alone. Moreover, gene expression profiling and bioinformatics analyses revealed that the MSC-exosome-β-TCP scaffold promoted bone regeneration mainly by activating the PI3K/AKT signaling pathway [133]. Exosomes released by hiPSC (hiPSC-Exos) combined with β-TCP scaffolds can potentially be used for bone repair. The β-TCP scaffolds incorporated into hiPSC-Exos were able to significantly promote bone regeneration in an osteoporotic rat skull defect model, and the therapeutic effect was increased with increasing concentrations of hiPSC-Exos. In addition to osteogenesis, the hiPSC-Exos + β-TCP scaffolds also markedly enhanced angiogenesis in the area of the calvarial defect, and the restoration of blood flow was capable of providing nutrients and renewable autologous cells to repair critical-sized bone defects [48]. BMSC-Exos-HIF1α loaded onto the β-TCP scaffolds implanted in the bone defect area repaired bone defects by promoting new bone regeneration in cranial critical-sized bone defect rats [134]. Additionally, β-TCP scaffolds are also employed to repair bone defects in alveolar regions for treating periodontitis. For example, exosomes secreted by SHED-derived exosomes combined with β-TCP scaffolds induced alveolar bone regeneration and neovascularization by promoting osteogenesis-related gene expression and phosphorylation of AMPK [135]. Exosomes released from human periodontal ligament stem cells (hPDLSCs) were loaded with β-TCP scaffolds and then applied to repair bone defects, leading to increased formation of alveolar bone in rat models of periodontitis [136].

### Bioactive ceramic supplemented with bone substitute

However, the limited bioactivity of ceramics may influence their regenerative effects. Therefore, bioactive materials such as bone substitutes can be added to promote their performance. Using a calcium sulfate-nanohydroxyapatite nanocement (NC) bone filler as the MSC-exosome carrier provides a newly improved approach for bone regeneration. After implantation in rat tibia critical defect models, this complex enhanced bone regeneration by inducing bone mineralization [137].

## Hydrogels

Currently, hydrogels scaffolds have been extensively utilized in the field of bone regeneration, as they are compatible biomaterials acting as exosome carriers and exosome delivery reservoirs. For bone regeneration, hydrogels may be synthesized from various biodegradable polymers, such as hyaluronic acid (HA), chitosan (CS), silk fibroin (SF), alginate (ALG) and polyethylene glycol (PEG) [138].

Hydrogels are three-dimensional network structure polymer chains with superior mechanical strength and can mimic the natural ECM of bone tissue. Therefore, hydrogels provide nutrient environments suitable for endogenous cell proliferation, thus presenting a prospective ability of exosome encapsulation for bone regeneration [139]. Due to the 3D network structure and physiochemical properties of the hydrogels, the encapsulated bioactive molecules are confined in the meshes. In addition, exosomes formulated in hydrogel scaffolds may reduce the degradation rate and easily maintain the release of exosomes as needed, contributing to the high local concentrations of desired pharmacologically important molecules contained in exosomes to achieve desirable therapeutic effects. These properties make hydrogels a promising scaffold for cell-free therapy in targeting bone tissue sites and facilitate localized delivery of therapeutic agents, thereby promoting bone regeneration for treating bone defects [138].

### Natural hydrogels

Hydrogels prepared by natural polysaccharides and proteins, including hyaluronic acid, hydrogel alginate, chitosan and gelatin, can be used as scaffolds for exosome carriers with desirable biodegradability and proper cell interactions in the treatment of bone defects.

HA is a nonimmunogenic natural polymer that is the major component of the ECM structure and critical to tissue regeneration [140]. Umbilical MSC-derived exosomes (uMSCEXOs) combined with HA hydrogels (HA-Gel) and then customized nanohydroxyapatite/poly-ε-caprolactone (nHP) scaffolds were shown to repair cranial defects in rats. The uMSCEXOs/HA-Gel/nHP complex markedly accelerated the bone regeneration process [141]. Yang et al. combined HA-ALG hydrogels and hydroxyapatite (HAP) with hucMSC-derived exosomes targeting bone defect sites in a calvarial defect rat model for bone regeneration. The composite hydrogel system durably retained exosomes at the targeting sites and significantly promoted the healing of damaged bone by enhancing osteogenic abilities [142]. CS is another polysaccharide with a linear structure composed of β-(1,4)-linked D-glucosamine and N-acetyl-D-glucosamine units with intrinsic bone healing properties. BMSC-derived exosomes were added to the CS/β-glycerophosphate hydrogel for bone regeneration in rats with calvarial defects. These exosome-release hydrogels were biocompatible and exhibited excellent therapeutic effects for repairing bone tissues [143]. Gelatin-based hydrogels have also attracted attention as potentially implantable materials in bone engineering applications due to their functionality. For example, hASC-derived exosomes combined with gelatin NPs hydrogel scaffolds accurately transported to the target sites and exerted a stronger bone repair capacity [70]. Natural hydrogel scaffolds have also shed light on the therapy of bone loss in periodontitis. For example, BMSC-derived exosomes were isolated and then loaded into a natural hydrogel for injection into rats with experimental periodontitis. The BMSC-Exo-hydrogel system promoted the migration, proliferation, and osteogenic differentiation of hPDLSCs for periodontal regeneration [62]. Exosomes derived from dental pulp stem cells (DPSCs) encapsulated into CS hydrogel effectively accelerated the regeneration of alveolar bone in a mouse model of periodontitis [144].

Moreover, the secretome of MSCs is able to provide specific peptides, and hydrogel-assisted exosomes combined with these peptides can improve the effects of bone regeneration. For example, small intestinal submucosa (SIS) hydrogels incorporated with 3-(3,4-dihydroxyphenyl) propionic acid (CA) modified by fusion peptides and exosomes derived from BMSCs were considered desirable for bone regeneration due to the positive effect of the exosomes in promoting the osteogenic differentiation of BMSCs. The combination of SIS hydrogels and CA significantly improved the mechanical properties of the hydrogels, and fusion peptides were designed to enhance the retention and stability of exosomes. Therefore, these scaffolds strengthen the therapeutic effect of MSC-derived exosomes on bone defects [145].

### Synthetic hydrogels

Despite the advantages of using natural hydrogels in bone repair, the drawbacks of natural materials for hydrogel preparation are less stability, and undesirable mechanical characteristics. The incorporation of MSC-derived exosomes into synthetic hydrogels may be used to improve bone regeneration effects. Hydrogels can be synthesized from various polymeric biodegradable materials. Synthetic hydrogels present a longer shelf life, reliable mechanical properties and lower risk of immunogenicity, making them suitable for bone tissue regeneration. For instance, stem cells from apical papilla-derived exosomes (SCAP-Exos) loaded into a bioresponsive PEG/DNA hybrid hydrogel facilitated bone regeneration for diabetic bone defects by the controlled release of SCAP-Exos [146]. A coralline hydroxyapatite/SF/glycol CS/difunctionalized PEG self-healing hydrogel was synthesized, and hucMSC-derived exosomes were loaded into the hydrogel. This synthetic hydrogel containing exosomes effectively promoted bone regeneration [147].

### Hydrogels combined with bone engineering materials

The use of hydrogels alone may cause problems, due to their poor mechanical and chemical properties. The emergence of composite bone engineering materials, such as metal–organic frameworks (MOFs) and β-TCP scaffolds, can promote the widespread use of hydrogel composite scaffolds in bone regeneration. MOFs are promising platforms for biomedical applications because of their structural diversity, high surface areas, adjustable porosity, simple surface functionalization and tunable biocompatibility. The application of MOF scaffolds as exosome carriers is a novel strategy for bone regeneration. For instance, hADSC-derived exosomes and Mg-gallic acid (GA) MOF were combined for bone regeneration applications, taking advantage of hADSC-Exos, Mg^2+^, GA and MOF scaffolds. As the scaffolds degraded, exosomes were released continuously and then internalized by hBMSCs to enhance their osteogenic effects by stabilizing the bone graft environment, promoting osteogenic differentiation and stimulating bone reconstruction. Furthermore, exosomes secreted from the composite scaffolds were also proven to induce new bone formation and osseointegration in the rat calvarial defect model [148]. β-TCP can also be combined with hydrogels to enhance bone regeneration. Zhang et al. extracted exosomes originating from rat BMSCs (rBMSCs) and then cocultured them with polyethylene glycol maleate citrate (PEGMC) hydrogels and β-TCP to promote bone regeneration. The PEGMC/β-TCP-MSC-Exos showed great potential in rapid osteogenesis [149].

## Synthetic polymers

Synthetic polymers with tunable mechanical properties, such as poly-lactic-co-glycolic acid (PLGA) and polylactic acid (PLA), have also been used as scaffolds for the development of biocompatible and biodegradable polymeric structures to regenerate bone tissues.

PLGA with excellent mechanical strength and biodegradation properties is another widely used biocompatible scaffold for bone regeneration. For instance, hASC exosomes immobilized on polydopamine-coated PLGA scaffolds effectively promoted bone healing in mouse skull defect models, and polydopamine (pDA) was used to provide a more efficient coating on the PLGA substrate. hASC-derived exosomes combined with PLGA/pDA scaffolds were capable of consistently releasing exosomes, resulting in enhanced bone regeneration through their osteoinductive effects. In addition, this exosome-composite scaffold also promoted MSC migration and homing in newly formed bone tissues [150]. PLGA and PEG were engineered with human DPSC (hDPSC)-derived exosomes to improve the controlled release of exosomes for bone regeneration. These engineered scaffolds constantly released osteogenic hDPSC-derived exosomes that facilitated the osteogenic differentiation of BMSCs, leading to mineralization and accelerated bone healing [151]. PLA is a versatile and biodegradable scaffold widely used in repairing tissue defects. Exosomes were isolated from MSCs and coated with porous 3D PLA scaffolds to potentiate osteogenic differentiation. PLA-MSC-Exos significantly improved osteogenesis, which holds great potential for applications in bone tissue regeneration [152]. Polyetheretherketone (PEEK) is a noncytotoxic, highly biocompatible and chemically stable aromatic polymer material that can be used for bone regeneration. BMSC-derived exosomes packaged with tannic acid-modified sulfonated PEEK continually released exosomes and exerted osteoimmunomodulation effects to enhance osteogenesis by promoting macrophage M2 polarization [153].

## Limitations and future perspectives

Despite the promising application of exosomes in bone regeneration, there are certain limitations with the use of MSC exosomes for bone regeneration [154]. The main bottleneck in clinical application is the lack of standardized isolation and purification strategies for exosomes. Exosomes, as carriers of elements of cellular communication and interaction, can be detected in nearly all biological fluids including blood, saliva, urine and cerebrospinal fluid [155]. Effective extraction and separation of exosomes from different sources for bone regeneration are challenging. Ultrafiltration is the most commonly used method to isolate exosomes, with the advantages of easy operation and screening based on size [156]. However, it still has problems with large-scale production. The production of both high-quality and high-quantity exosomes remains challenging for the translation of these nanosystems into the clinic [44, 157].

Although the therapeutic benefits of modifications and exosome engineering have been demonstrated in various bone diseases, some challenges in preconditioning methods may limit their applications. MSC-exosome preconditioning can promote the anti-inflammatory and/or osteogenic potential of exosomes by regulating the secretion and content of MSC exosomes. However, the optimal time and intensity of different preconditioning still need to be further explored because of the heterogeneity of MSCs from different sources [51]. In addition, the long-term effects of preconditioning on the properties of MSCs and the evidence of consistently obtaining desirable exosomes from engineering methods still need to be evaluated in clinical [158, 159]. Accordingly, addressing these problems may increase their possibility of regenerative applications.

The emergence of biomaterials provides various options for exosome-based therapy; encapsulating exosomes into biomaterial scaffolds can optimize their application in bone regeneration, but there are still some limitations. For instance, it is difficult to maintain exosome release by scaffolds. Although the slow release is achieved, strategies for the consistently stable release of exosomes are not available, and the most suitable release rate for bone regeneration has not yet been explored [160]. Additionally, there was insufficient evidence to support the scalable manufacturing of scaffolds and efficient delivery of exosomes, and thus, further studies need to be performed to address these issues [138]. The promising cell-free therapy will surely attract deeper investigations to improve the production efficiency and quality of exosomes, the preconditioning or engineering methods, and the release of exosomes from the matched scaffold at the most appropriate rate, thereby maximizing exosome function in bone regeneration.

It should be noted that there is still a long way before exosome-based therapy can be comprehensively applied for bone regeneration in clinical settings. Many hurdles should be overcome before the clinical translation of MSC-derived exosomes. Firstly, the loading efficiency for exosomes is relatively low as their naturally complex contents limit the space for the loading of exogenous therapeutics into exosomes [161, 162]. More effective modification methodologies are required to develop to improve the drug loading efficiency. Secondly, the original sources, physiological states and cultural conditions of MSC may impact the targeting and biological properties of isolated exosomes [163–165]. Therefore, widely applicable standards for the isolation of MSC-derived exosomes should be established. Thirdly, several factors, such as the dosage of MSC exosomes and the use of scaffolds, may influence the potency of MSC exosomes for bone regeneration. A particular source and concentration of exosomes, a specific scaffold or delivery route and the frequency of treatment need to be identified for optimization in different treatments of specific bone defect/disease indications [16]. Exosomes provide a new direction for bone defect treatments. However, the regenerative efficacy of MSC exosomes for treating bone defects is still in infancy, as the current research is limited to small animal models. Efforts to progress to clinically related large animal models and ultimately to clinical trials would likely be helpful to advance the field of MSC exosomes for bone regeneration.

## Conclusion

The process of natural bone healing lacks the capacity to repair massive bone defects beyond a critical size, which makes the treatment of bone defects an intractable clinical problem [166]. Currently, progress in the use of bone grafting for clinical applications has many challenges, such as invasive surgical procedures, pain, secondary complications and disease transmission. However, cell-free therapy has great potential for bone regeneration, with MSCs as the most commonly used cell-free source [167]. MSC-derived exosomes are able to promote bone regeneration, which is of great significance for the effective application of bone defect repair. Moreover, modifications of exosomes, exosomes engineering and combined with biomaterial scaffolds markedly enhance their therapeutic effects [57]. Although there are currently limitations in MSC-derived exosome-based cell-free therapy, this is a promising field in bone regeneration, which can attract further investigations to confirm the clinical effect of treating bone defects.

## Data Availability

Not applicable.

## Abbreviations

MSC: Mesenchymal stem cell
hASCs: Human adipose tissue-derived mesenchymal stem cells
CM: Conditioned medium
BMSC: Bone marrow mesenchymal stem cell
hucMSC: Human umbilical cord mesenchymal stem cell
VEGF: Vascular endothelial growth factor
MVBs: Multivesicular bodies
hiPSC: Human-induced pluripotent stem cells
HIF-1α: Hypoxia-inducible factor
SHEDs: Stem cells from human exfoliated deciduous teeth
hPDLCs: Human periodontal ligament cells
TNF-α: Tumor necrosis factor-alpha
GMSCs: Gingival tissue-derived mesenchymal stem cells
TRAP: Tartrate-resistant acid phosphatase
IL-1β: Interleukin-1β
TGF-β1: Transforming growth factor-β1
mBMSC: Mouse bone marrow mesenchymal stem cell
BMP2: Bone morphogenetic protein 2
NPs: Nanoparticles
STExos: Exosomes derived from bone marrow stromal cells
CXCR4: C-X-C motif chemokine receptor 4
hMSCs: Human mesenchymal stem cells
MBG: Mesoporous bioactive glass
β-TCP: β-Tricalcium phosphate
hiPSC-Exos: Exosomes released by human-induced pluripotent stem cells
hPDLSCs: Human periodontal ligament stem cells
HA: Hyaluronic acid
CS: Chitosan
SF: Silk fibroin
ALG: Alginate
PEG: Polyethylene glycol
ECM: Extracellular matrix
uMSCEXOs: Umbilical mesenchymal stem cell-derived exosomes
HA-Gel: Hyaluronic acid hydrogels
nHP: Nanohydroxyapatite/poly-ε-caprolactone
DPSCs: Dental pulp stem cells
SIS: Small intestinal submucosa
CA: 3-(3,4-Dihydroxyphenyl) propionic acid
SCAP-Exos: Stem cells from apical papilla-derived exosomes
MOFs: Metal–organic frameworks
GA: Gallic
PEGMC: Polyethylene glycol maleate citrate
PLGA: Polylactic-co-glycolic acid
PLA: Polylactic acid
pDA: Polydopamine
PEEK: Polyetheretherketone
